# Characterization and Hemostatic Potential of Two Kaolins from Southern China

**DOI:** 10.3390/molecules24173160

**Published:** 2019-08-30

**Authors:** Changjiao Gan, Hongjie Hu, Zhiyun Meng, Xiaoxia Zhu, Ruolan Gu, Zhuona Wu, Hongliang Wang, Donggen Wang, Hui Gan, Jinglin Wang, Guifang Dou

**Affiliations:** 1 Academy of Military Medical Sciences, Beijing 100850, China; 2 Zhengzhou Institute of Multipurpose Utilization of Mineral Resources, Zhengzhou 450006, China

**Keywords:** Wenchang kaolin, hemostatic agent, Maoming kaolin, clay

## Abstract

The physicochemical properties and potential hemostatic application of Wenchang kaolin and Maoming kaolin were inspected and evaluated. Chemical composition analysis, Fourier transform infrared (FTIR) spectroscopy, surface area determination, X-ray diffraction, particle size, scanning electron microscopy (SEM) observations, and zeta potential analysis were performed to quantify the physical and chemical properties of the two kaolins. The results showed that both kaolins have typical FTIR bands of kaolinite with a weight fraction for kaolinite over 90 wt%. Larger conglobate aggregates of Maoming kaolin demonstrated wider particle size distributions with two peaks at 3.17 and 35.57 μm, while the book-like Wenchang kaolin had narrow particle size distribution, with a frequent size of 5.64 μm. Furthermore, thrombelastography, the whole blood clotting tests (WBCT), plasma recalcification time (PRT) measurement, and MTT assay were performed to measure the clotting activities and biocompatibility of the two kaolins. The results showed that both kaolins could promote blood coagulation with good cytocompatibility, while Wenchang kaolin had a better procoagulant activity than Maoming kaolin. These findings demonstrated Wenchang kaolin to be a more suitable local source material for application as a hemostatic agent.

## 1. Introduction

Kaolin is a versatile clay that has been widely used for different products such as ceramics, coating, water treatment, pesticides, and substrate for catalysis [1,2]. In recent years, the application of kaolin has been expanded to the field of medicine as a powerful exogenous coagulation blood material [3]. The possibility of these applications depends on the geological conditions of kaolinites, as well as their mineralogy, chemical, and physical properties such as particle distribution, color, component, and potential characteristics [1,4].

Wounds caused during surgical operations, accidents or wars may produce uncontrolled massive bleeding leading to hemorrhagic shock and death. Ideal hemostatic agents should control massive hemorrhage rapidly, while being biocompatible, stable, easy to manufacture, and low cost. In recent years, the use of several inorganic materials has been reported for accelerating blood coagulation, including zeolite, bentonite, kaolin, porous silica, and smectite [5,6,7,8,9,10]. Zeolite adsorbs plasma and concentrates blood cells but hydration of zeolite is an exothermic reaction that can cause severe burns to tissue around the wound [11,12]. Smectite granules (e.g., WoundStat) show high hemostatic efficacy but the granules were shown to enter the systemic circulation and cause distal thrombosis in vital organs; the product is no longer recommended by FDA for tactical combat casualty care (TCCC) [13,14]. Kaolin powder dispersed in nonwoven medical gauze is approved by the USA-FDA and is currently the preferred packing to induce clotting in arterial wounds not amenable to tourniquet application to stem blood flow. In 2008, kaolin began replacing zeolite products (QuikClot^®^, QuikClot™, ACS™, ACS™+) in combat medical dressings and is marketed widely as QuikClot^®^ Combat Gauze (QCG, Z-Medica Corporation, CT). QCG has been used in successfully stopping the bleeding from serious wounds of liver, mesentery, and femoral vessels within a minute in large animal testing [10,15,16,17,18], achieving hemostasis with a complete efficiency of 84.8% in patients [19]. It is reported that kaolin promotes clotting by activating Factor XII, in which it initiates the intrinsic clotting cascade via activating Factor XI and ends with the formation of a fibrin clot. In addition, kaolin can also promote the activation of platelet-associated Factor XI to initiate the intrinsic clotting cascade normally in Factor XII-deficient patients [20].

There are several primary kaolin deposits found in China, especially in southern China. These kaolin deposits are derived by alterations in granitic rocks, volcanic rocks, and kaolinitic sands [21]. The presence of the only large sedimentary deposit near Maoming of western Guangdong Province, China, has been reported previously, which is actually a kaolinitic sand of Late Tertiary age [22]. Most of the recent scientific studies in China have focused on understanding the use of kaolin as a raw material for catalysis and adsorption [23,24,25,26]. Few studies have been conducted to understand the properties associated with raw material selection, processing, structure, and elemental composition, which are the key determinants for hemostatic application of kaolin [11]. Two kaolins from Wenchang in Hainan Province and Maoming in Guangdong Province were collected and characterized to determine their physical and chemical properties, as well as the in vitro coagulation activity and cytocompatibility. The purpose of the work was to analyze the characteristics of two kaolinites from southern China and establish their suitability for hemostatic application. This study might reveal the structural and surface properties of kaolinites that influence the blood-clotting response.

## 2. Results

### 2.1. Chemical Composition Analysis

The results of XRF analyses are shown in Table 1. SiO_2_ and Al_2_O_3_ were found to be the major chemical compounds of both Wenchang kaolin and Maoming kaolin [27]. The results showed that there were significantly higher contents of CaO, K_2_O, Fe_2_O_3_, Na_2_O, CeO_2_, and Cl in Wenchang kaolin than in Maoming kaolin. Here, the contents of MgO, CaO, and Fe_2_O_3_ may affect the hemostatic activity of the two kaolins.

### 2.2. FTIR

**Figure 1:**
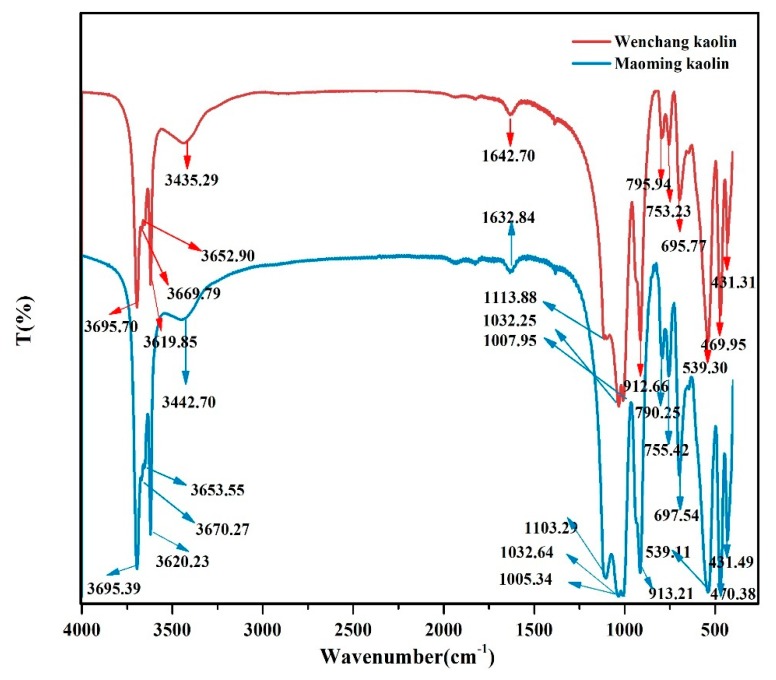
FTIR spectra corresponding to the Wenchang kaolin and Maoming kaolin samples.

The FTIR spectra in Figure 1 shows the typical bands of kaolinite. In the region between 3700 and 3600 cm^−1^, the stretching frequency of OH bands showed four stretching bands at 3620, 3653, 3670, 3695 cm^−1^ [28,29,30,31,32]. The peaks at 3435 cm^−1^ in the Wenchang kaolin and 3443 cm^−1^ in the Maoming kaolin signify the interlayer water OH stretching vibration, and the peaks at 1643 cm^−1^ in the Wenchang kaolin and 1633 cm^−1^ in the Maoming kaolin pinpoint the physical adsorption of water OH vibration. Bands at 796 cm^−1^ and 753 cm^−1^ of Wenchang kaolin, together with 790 cm^−1^ and 755 cm^−1^ of Maoming kaolin are attributed to (Al–OH) hydroxyl groups vertical to the surface. In the spectrum between 1000 and 1120 cm^−1^, corresponding to Si–O stretching modes, there were observed bands at 1114, 1032, and 1008 cm^−1^ in the Wenchang kaolin, and at 1103, 103,3 and 1005 cm^−1^ in the Maoming kaolin. The band at 912 cm^−1^ represents Al–Al–OH bonds [2], and the band at 539 cm^−1^ represents the Al–O–Si bending vibration [33].

### 2.3. XRD Analysis

**Figure 2:**
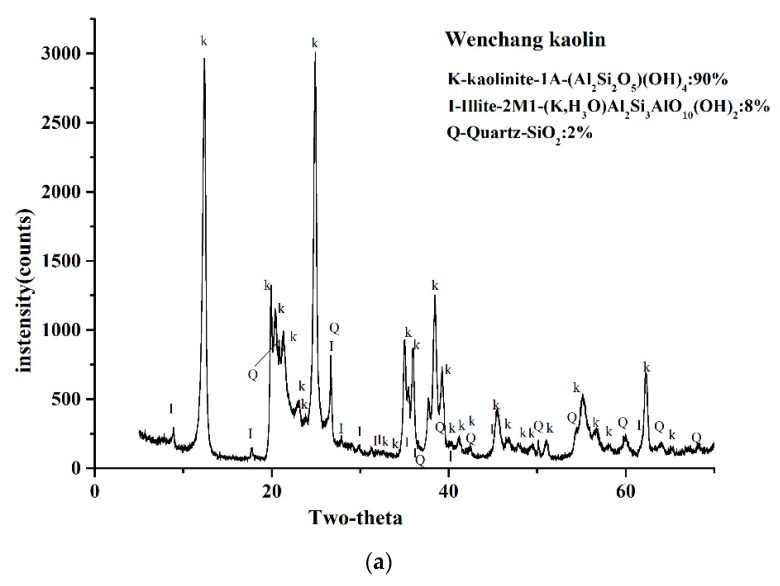

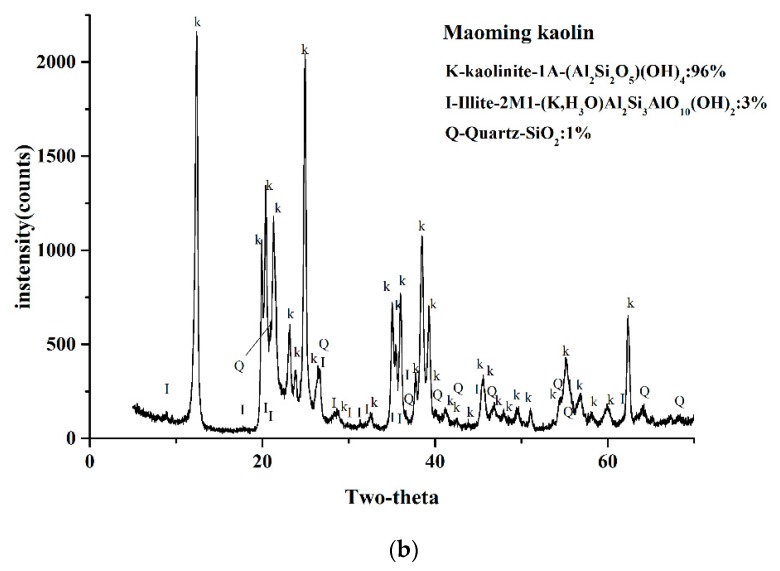
X-ray powder diffraction patterns of the two kaolinites.

Figure 2 displays the XRD results of the two kaolin powders in the range of 2θ = 5–65°. The results showed that in the two kaolin XRD patterns, both kaolinites had strong reflection, and a few reflections can be attributed to illite and quartz. By referring to the ICSD calculation data card of the minerals, quantification of the weight fraction indicated that the kaolinite weight fraction of Wenchang kaolin was 90 wt%, illite was 8 wt%, quartz was 2 wt%, while the weight fraction of kaolinite in Maoming kaolin was 96 wt%, the illite content was 3% by weight, and quartz was 1% by weight.

### 2.4. Particle Size Distributions

**Figure 3:**
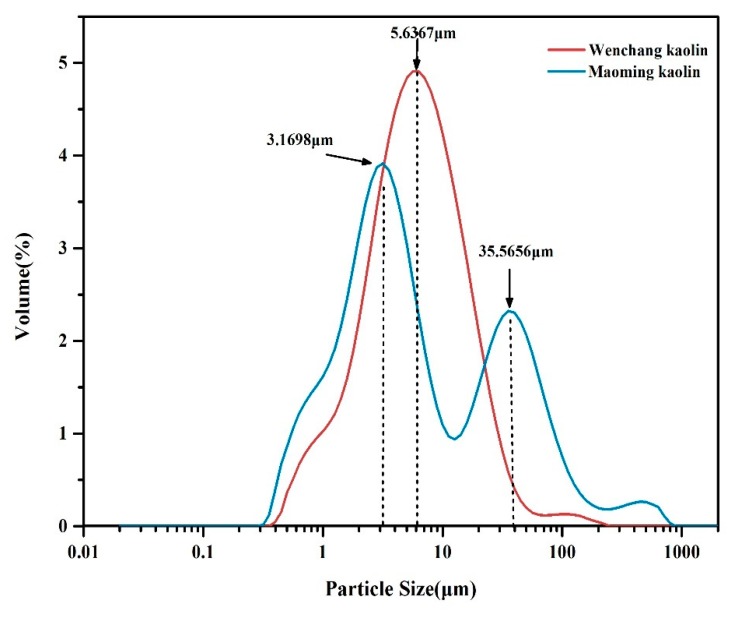
Particle size distribution of the two kaolins.

The particle size distributions of Wenchang kaolin and Maoming kaolin are displayed in Figure 3. The results showed that the particle size of Wenchang kaolin ranged from about 0.40 μm to 224.40 μm and most of the particles were less than 20 μm. However, the particle size of Maoming kaolin ranged from 0.32 μm to 796.21 μm, presenting a much wider size distribution than Wenchang kaolin. Data of the particle size index for the two kaolinites showed that the particle size cumulative volume frequency (D_0.9_) of Maoming kaolin was 57.61 ± 2.16 μm, indicating larger grain diameter than Wenchang kaolin with a D_0.9_ of 17.75 ± 0.53μm. In addition, the main frequent particle sizes of Maoming kaolin were 3.17 μm and 35.57 μm, and the two peaks of the particle size distributions that emerged in the curve of Maoming kaolin are presented in Figure 3. Only one peak was displayed in the particle size distribution of Wenchang kaolin, with the most frequent size of 5.64 μm.

### 2.5. Surface Area Determination

The surface area characteristics of the two kaolinites were determined by nitrogen sorption isotherms (Table 2). The t-Plot external and BET surface area were similar between Wenchang kaolin and Maoming kaolin. The pore volume and the diameter of Maoming kaolin were a little larger than those of Wenchang kaolin. The whole blood clotting times (WBCT) of Maoming kaolin (3.88 min) was not faster than that of Wenchang kaolin (2.88 min), although its pore volume (0.11 cm^3^/g) and diameter (24.87 nm) were somewhat larger than those of Wenchang kaolin (0.07 cm^3^/g and 19.04 nm).

### 2.6. Kaolinite Micromorphology and Microtexture

**Figure 4:**
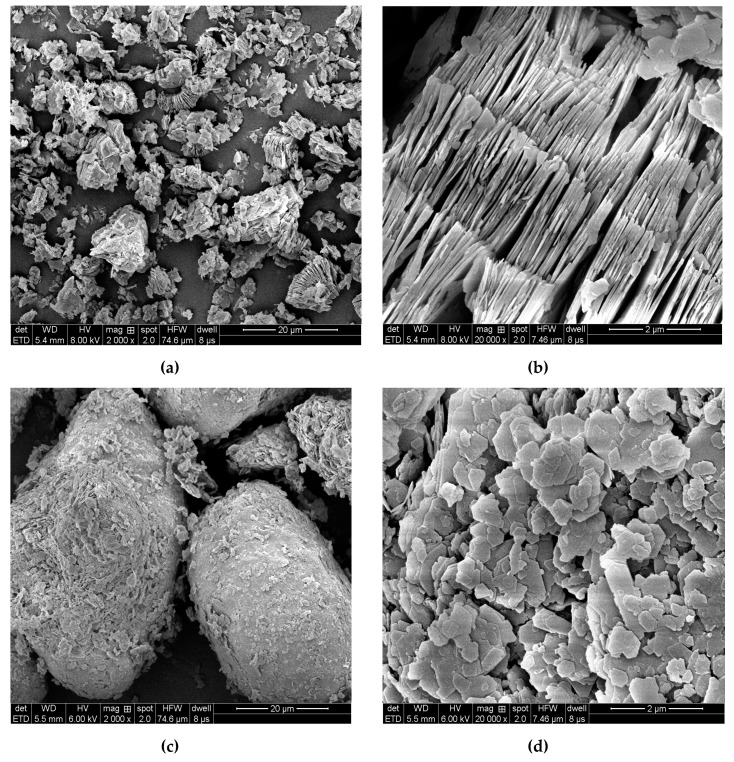
Field emission scanning electron microscopy (FESEM) images of Wenchang kaolin (**a**,**b**) and Maoming kaolin (**c**,**d**).

In the kaolin micrographs obtained by FESEM (Figure 4), the form and size of the particles were estimated. Both Wenchang kaolin and Maoming kaolin microscopically exhibited a typical pseudohexagonal shape; however, they displayed diverse morphologies and diameters. The layered structure of Wenchang kaolin demonstrated staked layers and booklet morphology (Figure 4a,b). The flat layer structure was similar to that of the typical morphology of kaolinite, which reported that the possible plate thickness is in the range of 100–150 nm [31,34] Maoming kaolin displayed a different particle size distribution, which exhibited an amount of large kaolinite particle agglomerates (Figure 4c,d). This kind of conglobate structure might be due to the strong interaction between the kaolinite charge and the surface charge of the impurities in the ore [2]. SEM images indicated that the two clays have diverse morphologies and diameters. Microscopically, the book-like Wenchang kaolin had densely packed layers, while the Maoming kaolin had larger conglobate aggregates, indicating the variations in the genesis of the two kaolins.

### 2.7. Zeta Potential Analysis

**Figure 5:**
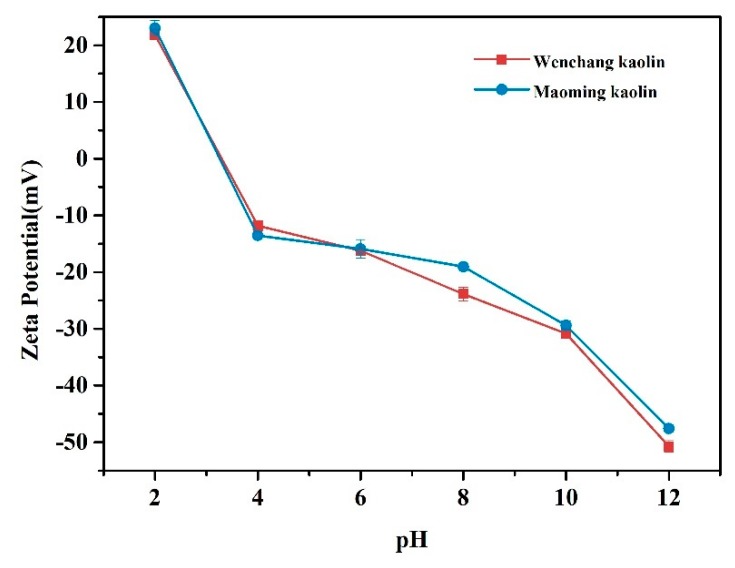
Zeta potential of kaolin samples with varying pH.

The zeta potentials for both kaolin samples in the pH range 2–12 are shown in Figure 5. At blood pH of 7.35–7.45, the surface charge value that was obtained between pH 6–8 should be specifically focused. The results showed that both kaolinites displayed negative potentials in the pH range 4–12. Wenchang kaolin showed more negative surface charge than Maoming kaolin between pH 6 and 8. Between pH 6 and 8, the zeta potential of Wenchang kaolin was −16.2 ± 0.2 mV to −23.9 ± 1.2 mV, and that of the Maoming kaolin was −15.9 ± 1.6 mV to −19.0 ± 0.5 mV.

### 2.8. Thrombelastograph Measurements of Clotting Agents in Whole Blood

**Figure 6:**
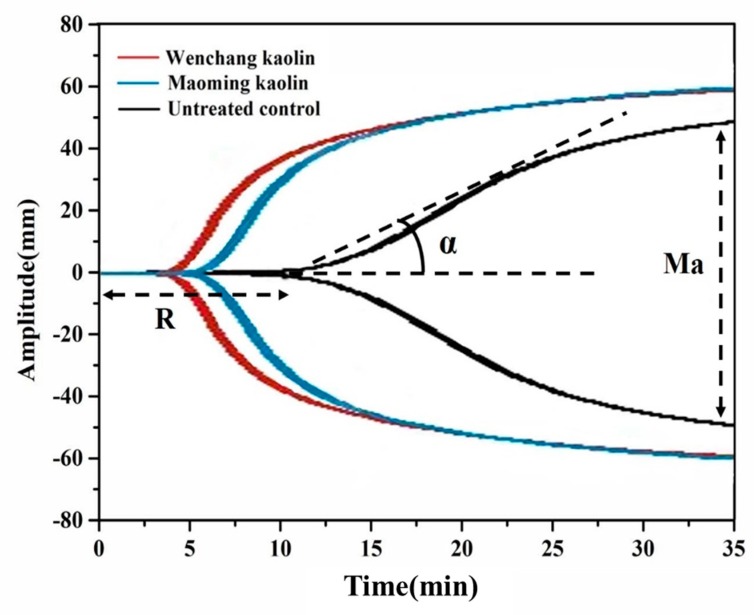
TEG showing the clotting characteristics of the Wenchang kaolin sample, Maoming kaolin sample, and untreated control in whole blood of beagle dogs.

Thrombelastography (TEG) was used to determine the clotting activities of the two kaolin samples in the whole blood of beagle dogs, which provided accurate data of initial clot initiation (R), rate of thrombus formation (α), and strength of the clotting (MA). As shown in Figure 6, the blood clotting activities with Wenchang kaolin suspensions and Maoming kaolin suspensions were highly increased compared to that of untreated controls. In our previous experiments, treatment of blood with the two kaolin powders initiated faster and stronger clot formation, resulting in tight TEG clotting curves. The two kaolin suspensions were used here instead of kaolin powders to reduce the dosage of kaolin. Albeit to a lesser extent, exposure to kaolin suspension still promoted faster and stronger hemagglutination than untreated controls. Meanwhile, the shorter blood clotting time (R) of Wenchang kaolin when compared with R of Maoming kaolin indicated that Wenchang kaolin activated coagulation more powerfully than Maoming kaolin. This showed that the two kaolin suspensions at relatively modest concentrations could also accelerate the coagulation process by activating Factor XII, leading to a significant increase in the speed of blood clot formation over untreated blood.

### 2.9. In Vitro WBCT

**Figure 7:**
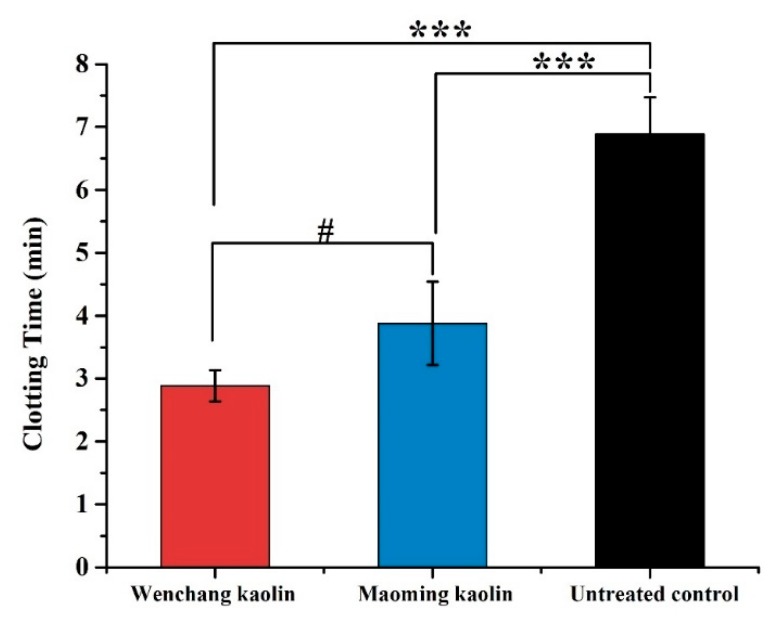
WBCT of two kaolins and untreated control (*n* = 5). (*** indicates extraordinary difference with untreated control and *p* < 0.001, # indicates significant differences between the WBCT of the two kaolins and 0.01 < *p* < 0.05).

The blood clotting times of Wenchang kaolin and Maoming kaolin in vitro are shown in Figure 7. Similar to the TEG results, the WBCT data also showed that the blood clotting time of Maoming kaolin (3.88 ± 0.66 min) was shorter than the untreated control (6.89 ± 0.58 min, *p* < 0.001). Meanwhile, the blood clotting time of Wenchang kaolin (2.88 ± 0.25 min) was much faster than Maoming kaolin (*p* < 0.05), showing statistically significant differences, and was extraordinarily shorter than the untreated control (*p* < 0.001). This indicated that Wenchang kaolin had a better procoagulant activity than Maoming kaolin.

### 2.10. Plasma Recalcification Time Measurement

PRT is commonly employed as an assessment index, signing the time required for fibrin clot formation when the calcium is replenished in the anticoagulated plasma.

**Figure 8:**
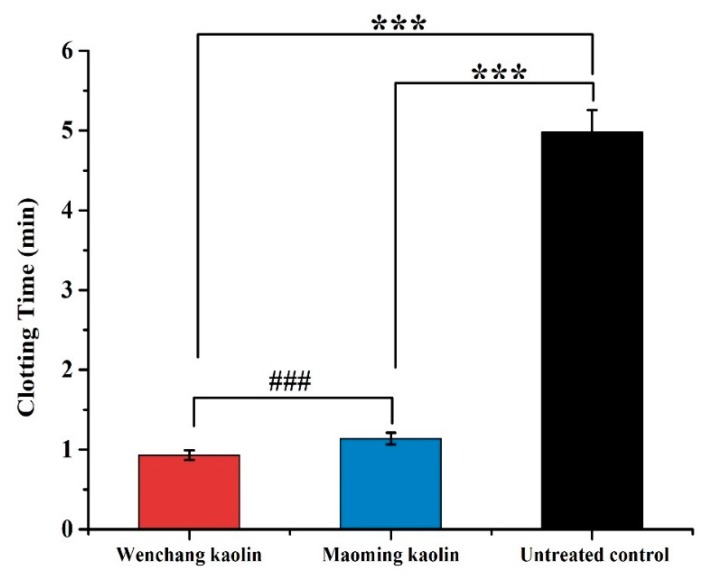
Plasma recalcification time (PRT)_of the two kaolin samples and the untreated control (n = 6). (*** indicates extraordinary difference with untreated control and *p* < 0.001, ### indicates extraordinary difference between the PRT of two kaolins and *p* < 0.001).

Figure 8 displays PRT values of Wenchang kaolin and Maoming kaolin. The data showed that the PRT of both Maoming kaolin (1.14 ± 0.08 min) and Wenchang kaolin (0.93 ± 0.06 min) were remarkably shorter than the untreated control (4.98 ± 0.28 min, *p* < 0.001). Furthermore, the PRT of Wenchang kaolin was faster than Maoming kaolin (*p* < 0.001), showing statistically significant difference. The contact activation of intrinsic cascade in the plasma could vary with the type of the surface.

### 2.11. Cell Viability Assay

**Figure 9:**
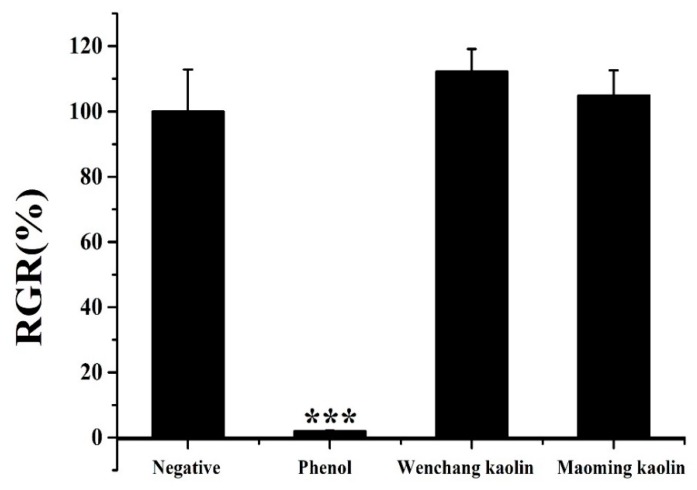
Biocompatibility of kaolin samples using MTT assay (n = 3). (*** indicates extraordinary difference with untreated control and *p* < 0.001).

Figure 9 displays the relative growth rate (RGR%) of L-929 cells with kaolin extractants and controls after culturing for 48 h. The relative viability of the phenol group was only 1.99 ± 0.14% when compared with the negative control group, which meant high cytotoxicity. Both Wenchang kaolin (with an RGR% value of 112.28 ± 6.84%, *p* > 0.05) and Maoming kaolin (with an RGR% value of 104.89 ± 7.67%, *p* > 0.05) showed noncytotoxicity and good biocompatibility against L-929 fibroblast cells after 3 days of seeding.

## 3. Discussion

Wenchang kaolin demonstrated faster clotting activity than Maoming kaolin. It is known that the contents of Ca^2+^ and Mg^2+^ cations might affect their coagulation kinetics because of certain enzymatic reactions in the clot cascade that require Ca^2+^ and Mg^2+^ cations. In particular, Ca^2+^ cations are a key factor in promoting blood coagulation, and the CaO content plays a role in blood clot initiation [35,36]. Intravenous delivery of Mg^2+^ cations has also been developed as a candidate therapy in patients with prolonged coagulation [37]. Fe_2_O_3_ can facilitate RBC aggregation and clotting [18] and Fe_2_O_3_ nanoparticles have been used for tissue repair [38]. The MgO contents of the two samples were similar, but the CaO and Fe_2_O_3_ contents were higher in Wenchang kaolin than Maoming kaolin, which might act as an influential factor on blood clotting activation.

The FTIR spectra and XRD analysis showed that both were typical kaolinites, with a weight fraction of kaolinite of over 90% and small parts of illite and quartz, while the quantity of illite was different. The particle size, shape, and distribution are considered as important physical properties and are closely correlated with the application of clay minerals [39]. Results of particle size analysis indicated that Wenchang kaolin might be more suitable for medical application than Maoming kaolin because of its narrow and uniform particle size distribution.

For some inorganic minerals, such as zeolite, it was reported that the larger external clay BET surface area and pore volume might speed up the blood coagulation, because of its correspondingly increased water absorption, which concentrated the blood and accelerated the coagulation [12,14,40]. Zeolites exhibit high BET surface areas (98–119 m^2^/g) and high total pore volumes (0.19–0.220 cm^3^/g) [41], which in turn can rapidly absorb water at the site of bleeding in a nonchemical reaction, effectively concentrating the platelets and clotting factors to promote coagulation [12]. These results further confirmed that the main hemostatic mechanism of kaolinite does not rely on water absorption to concentrate blood, which is different from zeolite [11]. This suggests that the clay BET surface area and pore volume of kaolin may slightly influence its hemostatic performance.

Natural aluminosilicate clay has been widely used as hemostatic wound dressings. The current generation of clay-based topical hemostatic wound dressings almost exclusively contains kaolin. Kaolin, in contact with plasma, can trigger the activation of the intrinsic blood clotting cascade by binding to the positively charged amino acids present in coagulation Factor XII (Hageman factor) via the negatively charged surface of kaolin. This binding might result in subsequent conformational changes in FXII, further giving rise to the generation of active FXIIa by autoactivation. FXIIa has been confirmed to directly contribute to fibrin formation. Fibrinogen is a soluble plasma glycoprotein which can be converted to insoluble fibrin by thrombin during blood clot formation [42,43,44,45]. To make space for the growth of connective tissue cells and wound healing, the fibrin must be removed by the proteolytic system. The expression of sulfhydryls on cancer cell membranes can cause the exchange of disulfides between the polypeptide chains of fibrinogen, which results in the formation of a fibrin-like polymer (called parafibrin). Due to the presence of hydrophobic bonds in its structure, parafibrin is completely resistant to proteolytic degradation and forms a shell on the surface of tumor cells, protecting them from destruction by phagocytic cells, which is different from fibrin. Selenium is an essential trace element that occurs in nature in two inorganic forms, as selenite (Se^4+^) and selenate (Se^6+^), and in a number of their organic derivatives. Se^4+^ reacts with the -SH groups of proteins to prevent the formation of parafibrin on tumor cells, and thus may play a role in the treatment of cancers [46,47,48]. Kaolin can trigger conversion of XII to XIIa and then contribute to fibrin formation, suggesting that kaolin and its derivatives may also be used for modulation and control of the response to treat cancer and other diseases in future biomedical applications [3].

PRT is an indicator of endogenous coagulation cascade activation, and acts as an important marker for biomaterial-induced coagulation activation [49]. TEG results showed that the two kaolins significantly speed up the initial fibrin formation compared to untreated blood, and Wenchang kaolin-activated blood clotting was more powerful than that of Maoming kaolin with less blood clotting time (R). Furthermore, the WBCT and PRT were applied as indicators of material-induced coagulation activation. The data showed that WBCT and PRT of Wenchang kaolin and Maoming kaolin were remarkably shorter than the untreated control (*p* < 0.001), and Wenchang kaolin was significantly faster than Maoming kaolin (*p* < 0.05). This indicated that Wenchang kaolin had a better procoagulant activity than Maoming kaolin. In vitro cytocompatibility tests showed that both clays were not cytotoxic and had good cytocompatibility for L-929 fibroblasts. This suggested that the two kaolins are safe, nontoxic and suitable for biomedical applications.

## 4. Materials and Methods

### 4.1. Materials

#### 4.1.1. Preparation and Analysis of the Clay Minerals

The kaolins used in this study were collected from Wenchang (19°20′ N; 108°21′ E) of Hainan province and Maoming (22°42′ N; 111°41′ E) of Guangdong province in China. The clay minerals used in this study were provided by Zhengzhou Institute of Multipurpose Utilization of Mineral Resources. Both Wenchang and Maoming kaolins were ground to acquire appropriate powders. For each clay powder, cyclone and hydraulic classification was applied, and then the feldspar and quartz sand were sieved out. Finally, the two powders were dried for 1 h at 300 °C and screened with 100 mesh. Particles below 150 microns were selected for the following analysis.

#### 4.1.2. Experiment Materials

RPMI 1640 medium and fetal bovine serum (FBS) were purchased from Gibco (Thermo Fisher Scientific, Waltham, MA, USA). Penicillin and streptomycin were purchased from North China Pharmaceutical Co., Ltd (Shijiazhuang, Hebei, China). NCTC clone 929 (L cell, L-929, derivative of Strain L) was obtained from the Cell bank of Chinese Academy of Sciences (Shanghai, China). Dimethyl sulfoxide (DMSO) and phenol were of analytical grade. Thiazolyl blue tetrazolium bromide (MTT) was purchased from AMRESCO (Houston, Texas, USA). Deionized water was prepared in our own laboratory.

### 4.2. Chemical Composition Analysis

The chemical compositions of the samples were measured by X-ray fluorescence (XRF) spectrometer (ARL ADVANT XP+, Thermo Fisher Scientific, Waltham, MA, USA) operating at 50 kV and 50 mA with a Lawrencium target [1].

### 4.3. Fourier Transform Infrared (FTIR) Spectroscopy

Nicolet 6700 Fourier transform infrared spectrometer (Thermo Fisher Scientific, Waltham, MA, USA). was used to measure the infrared spectra of the clays The spectra were then recorded in a wavenumber range from 400 to 4000 cm^−1^ with 32 scans per spectrum and a resolution of 4 cm^−1^ [2].

### 4.4. X-ray diffraction (XRD) Measurements

X-ray powder diffraction of the two kaolin samples was performed on an Xpert Pro MPD diffractometer (XRD, Malvern Panalytica, Eindhoven, Netherlands), which was operated at 40 kV and 40 mA with a Cu Kα (λ = 1.5406 Å) radiation. The samples were air desiccated and placed on the spinner of XRD, followed by scanning for 2θ ranging from 5° to 90° with a scan rate of 4 °/min and a step size of 0.02 [2,50]. Mineral compositions of the two clays were identified with JADE6.5 software. Inorganic Crystal Structure Database (ICSD) and Rietveld analysis were adopted to obtain the relative fractions in different phases for the two samples.

### 4.5. Particle Size Distribution

Particle size distribution was analyzed on Malvern Mastersizer 2000 (Malvern, UK) [1,2]. All samples were sonicated for 30 min before measurement. Each sample was tested thrice and expressed as mean values. The particle size characteristics included the average particle size (D_0.5_) and the particle size cumulative volume frequency (D_0.1_, D_0.9_).

### 4.6. Surface Area Determination

A Micrometrics ASAP 2460 (Norcross, Georgia, USA) was used for nitrogen gas sorption analysis of clay surface areas [11,51]. Prior to the surface area measurement, clays were dried at 200 °C for 12 h under nitrogen. Data were calculated using MicroActive 2.01 analysis (Norcross, Georgia, USA).

### 4.7. Scanning Electron Microscopy (SEM) Analysis

A scanning electron microscope (Quanta FEG 250, Hillsboro, Oregon, USA) was employed to observe the morphology of the clays [1,4]. The kaolin powders were sputtered with a thin layer of platinum to increase their electrical conductivity on a Leica EM SCD 500 at 16 mA with 120 s before field emission scanning electron microscopy (FESEM) analysis.

### 4.8. Zeta Potential Analysis

Prior to the analysis, a series of aqueous solutions in the pH range 2–12 were prepared [2,18]. Kaolin samples were dispersed at a concentration of 0.1 mg/mL in the aqueous solutions. The measurements were made in polystyrene cuvettes on a Malvern Zetasizer Nano S90 (Malvern, United Kingdom). Each measurement was made in triplicate at 25 °C using monomodal analysis and 12 cycles of runs. Between each sample, the dip cell electrode was rinsed and sonicated in deionized water for 3 min.

### 4.9. Thrombelastograph Measurements of Clotting Agents in Whole Blood

A Hemoscope Thrombelastrograph™ (TEG Hemostasis Analyzer 5000, Haemoscope, Niles, IL, USA) was chosen to assess the coagulation activity of the clays investigated [52,53]. It can provide quantitative data including the reaction time until clot formation (R), the rate of thrombus generation (α) and the maximum amplitude (MA) by measuring the torsion around a wire during blood clotting period.

Firstly, 1 mL fresh citrate-stabilized whole blood of beagle dog was prepared in each plastic vial, followed by immediate addition of 20 μL (0.1 mg/mL) of each kaolin suspension, and then gentle inversion of the mixture. Next, 20 μL of CaCl_2_ (0.2 M) was placed in a plastic sample cup and then heated to 37 °C. Finally, 340 μL whole blood mixed with kaolin suspension was added to the sample cup, which was loaded into position for beginning the test. The citrate-stabilized whole blood without kaolin treatment was tested as blank control. Five beagle dogs were used in this experiment.

### 4.10. In Vitro Whole Blood Clotting Tests (WBCT)

Five milligram clay samples were placed in flat-bottom glass vials and preheated at 37 °C. Then, 1 mL fresh whole beagle dog blood was slowly added into each vial and gently inverted for 3 s. The time was checked immediately after adding the whole blood. The vials were incubated at 37 °C and tilted every 10 s for observation. WBCT was performed to measure the time when the blood was totally coagulated. The blood sample without any addition of clay was tested in parallel as the untreated control group [54]. Five parallel tests were performed for each sample by using five beagle dogs in this experiment. All animal experiments were done with the permission of Institute of Animal Care and Use Committee (IACUC) at the Academy of Military Medical Sciences (AMMS). The ethical approval number was IACUC of AMMS-13-2017-017. IACUC of AMMS-13- 2016-017.

### 4.11. Plasma Recalcification Time (PRT) Measurement

Fresh New Zealand rabbits’ blood was collected and mixed with sodium citrate solution in tubes immediately. The anticoagulated whole blood was then subsequently centrifuged at 3000 rpm for 15 min, and the upper plasma was platelet-poor plasma (PPP). Each test tube with 0.1 mL PPP was prepared, and 0.1 mL of kaolin in suspension at a concentration of 1 mg/mL was joined and mixed quickly. Normal saline was used as a negative control to kaolin. Each tube was incubated at 37 °C for 3 min, followed by the addition of 0.1 mL CaCl_2_ solution to start plasma recalcification measurement. The time was recorded as the PRT when silky fibrin appeared in the mixture, which denoted clot formation [44]. The experiment was repeated 6 times and an average value was obtained.

### 4.12. Cell Viability Assay

The MTT method was universally employed for evaluating material biocompatibility [18,55,56]. L-929 mouse fibroblast cells were cultured in RPMI 1640 medium with 10% FBS at 37 °C. The kaolin samples were sterilized via Co_60_ gamma irradiation at a dose of 25 kGy and incubated in RPMI 1640 medium at 37 °C for 24 h. Then, the extractants of the samples incubated were gathered after centrifugation.

L-929 cells at a density of 5 × 10^3^ cells per well were spread in a 96-well plate and incubated for 24 h for cell attachment. Then, the extractants of the kaolins replaced the culture medium to be incubated with the cells for 48 h. After interaction, MTT solution was used for another 4 h exposure. After that, the extractant medium was drawn out and 150 μL DMSO was filled to dissolve the formazan crystals. An ELISA reader (SpectraMax 190) was used to measure the absorbance of formazan solution at 490 nm. The negative control was cells in the fresh culture medium, and the positive control was cells in the culture medium that contained 0.30% phenol. Cell viability was denoted with relative growth rate (RGR%), which was calculated by using the Equation (1):

RGR (%) = A_sample_/A_negative_ × 100% (1)

where A_sample_ represents absorbance of samples and A_negative_ represents negative control.

### 4.13. Data Analysis

Data was expressed as mean ± SD (standard deviation). The differences between experimental groups were compared by Student’s *t*-test. A *p*-value of less than 0.05 (95% level) was considered to be statistically significant.

## 5. Conclusions

Based on our analysis, both Wenchang and Maoming kaolin were typical kaolinites with good hemostatic activity, and Wenchang kaolin is better than Maoming kaolin. They could be applied for controlling bleeding following traumatic injury as locally sourced materials in China, and be developed as a positive activator for a TEG detection kit. This study also suggests that the MgO, CaO, and Fe_2_O_3_ contents, particle size and distribution, and zeta potential of the kaolin may influence its hemostatic performance. Cytotoxicity evaluation in vitro highlighted that both kaolins have good biocompatibility for tissue cells. Some kaolin-based hemostatic agents have been developed to prevent massive blood loss, contributing to making the hemostatic process easier and shorter. To further demonstrate the clinical potential of kaolin-based composites, future studies will be conducted on hemorrhage models, which will produce further scientific evidence for the use of kaolin-based composites for bleeding control.

## Figures and Tables

**Table 1:** Chemical compositions of two kaolin samples (wt%).

Oxides	Wenchang Kaolin	Maoming Kaolin
SiO_2_	46.950	46.960
Al_2_O_3_	35.960	36.410
K_2_O	1.570	0.664
Fe_2_O_3_	1.070	0.784
TiO_2_	0.327	0.407
Na_2_O	0.236	0.134
Cl	0.219	0.016
CaO	0.089	0.053
CeO_2_	0.045	0.014
P_2_O_5_	0.044	0.318
La_2_O_3_	0.038	0.022
SO_3_	0.036	0.176
MgO	0.033	0.034
Nd_2_O_3_	0.021	0.004
ZrO_2_	0.015	0.006
ThO_2_	0.014	0.001
Rb_2_O	0.013	0.004
Ga_2_O_3_	0.009	0.008
Y_2_O_3_	0.008	0.002
Pr_6_O_11_	0.004	0.003
Nb_2_O_5_	0.004	0.001
ZnO	0.004	0.003
MnO	0.004	0.003
PbO	0.003	0.003
IrO_2_	0.002	0.002
GeO_2_	0.002	0.001
V_2_O_5_	0.001	0.007
NiO	0.001	0.002
Sc_2_O_3_	0.001	0.004
MoO_3_	-	0.011
Cr_2_O_3_	-	0.003
^a^ LOI	13.28	13.95

^a^ LOI: loss on ignition at 980 °C.

**Table 2:** Surface area characteristics calculated from nitrogen sorption isotherms and comparison with clotting times in whole blood of beagle dogs.

Materials	BET Surface Area (m^2^/g)	T-Plot External Surface Area (m^2^/g)	Pore Volume (cm^3^/g)	Pore Diameter (nm)	Blood Clotting Time (min)
Wenchang kaolin	15.18	18.65	0.07	19.04	2.88 ± 0.25
Maoming kaolin	17.28	18.00	0.11	24.87	3.88 ± 0.66
